# *Ascaris lumbricoides* antigen exposure modulates T cell activation via regulation of IL-15Rα expression, STAT5 phosphorylation, and promotes differentiation of BCL6^low^ B cells

**DOI:** 10.3389/fimmu.2026.1766483

**Published:** 2026-02-23

**Authors:** Giggil Pushpamithran, Daniel Appelgren, Robert H. Gilman, Robert Blomgran

**Affiliations:** 1 Division of Inflammation and Infection, Department of Biomedical and Clinical Sciences, Faculty of Medicine and Health Sciences, Linköping University, Linköping, Sweden; 2 Division of Diagnostics and Specialist Medicine, Department of Health, Medicine and Caring Sciences, Faculty of Medicine and Health Sciences, Linköping University, Linköping, Sweden; 3 Laboratorio de Investigación en Enfermedades Infecciosas, LID, Universidad Peruana Cayetano Heredia, Lima, Peru; 4 Department of International Health, Johns Hopkins School of Public Health, Baltimore, MD, United States

**Keywords:** helminth infection, IL-15 signaling, immune modulation, inflammation, MZ B cells, STAT5 phosphorylation, T cell activation, tuberculosis

## Abstract

**Background:**

*Mycobacterium tuberculosis* (Mtb) causes active tuberculosis (TB) in approximately 10 million people annually, resulting in 1.6 million deaths. TB and helminthiasis have a significant geographical overlap, and helminth infections can trigger immunological responses that dampen Th1 immunity, which is essential for Mtb containment. While B cells are known to modulate T cell responses, their role during helminth/TB co-infection remains unclear.

**Objective:**

This study aimed to analyze the effect of helminth exposure on T cell activation in a T and B cell co-culture system.

**Methods:**

T and B cells were isolated from healthy donor blood, stimulated with aCD3/aCD28, and exposed to *Ascaris lumbricoides* protein antigens (ASC) or *Schistosoma mansoni* soluble egg antigen (SM).

**Results:**

B cells reduced T cell proliferation, and SM exposure partly attenuated this inhibitory effect on CD4 T cells. ASC exposure did not affect T cell proliferation but increased soluble IL-15Rα and surface IL-15Rα and IL-2/15Rβ on CD4 T cells. Restimulation with recombinant human IL-15 revealed reduced and unsustained STAT5 activation in CD4 and CD8 T cells in ASC-exposed co-cultures. Additional analysis showed altered phosphorylation of STAT1, STAT3, and STAT6, indicating broader impairment of IL-15 responsiveness and a dampened Th1 activation profile. BCL6/BLIMP-1 transcription factor expression of B cells in ASC-exposed co-cultures suggested a shift toward a more differentiated phenotype, such as plasma cell, memory B cells, or marginal zone MZ B cells, which are all typically BCL6^low^. The *in vivo* analysis of peripheral blood mononuclear cells from individuals in a helminth/TB endemic region showed increased frequencies of CD38^hi^CD24^hi^ B regulatory cells in helminth infected, and MZ B cells (IgM^high^ unswitched B cells) in both helminth-infected and helminth/TB co-infected individuals.

**Conclusion:**

ASC antigen exposure modulates T cell activation through dynamic regulation of the IL-15-pathway and STAT signaling. These findings suggest that ASC exposure may help prevent prolonged IL-15-driven responses, potentially limiting excessive inflammation during TB. B cells in ASC-exposed co-cultures exhibit transcriptional changes consistent with a shift away from the germinal center phenotype toward more differentiated states.

## 1 Introduction

It is estimated that one-quarter of the world’s population is latently infected with *Mycobacterium tuberculosis* (Mtb), and that about 10 million people develop active tuberculosis (TB) yearly (1). There is a significant geographical overlap between TB and helminth infections (2), with over one billion individuals infected with soil-transmitted helminths (3). Helminth/TB co-infection can have significant implications for the immune response to Mtb and disease progression (4).

Intestinal helminths can significantly modulate the protective Th1 responses of CD4+ T cells, which are crucial for Mtb containment by activating macrophages and enhancing their microbicidal capacity (5, 6). However, to effectively combat Mtb, a balanced immune response is crucial to control bacterial replication and regulate inflammation, preventing tissue damage and transmission. Proper regulation of inflammation is essential, as excessive inflammation can impair immunity, cause lung damage, and enhance transmission. The Th2 response induced by helminths can promote expansion of regulatory T cells (Tregs) with the ability to suppress both Th1 and Th2 responses, facilitating persistence of Mtb and helminths (7).

B cells play a crucial role in the immune response against Mtb (8), including antibody and cytokine production, antigen presentation, and interactions with other immune cells (9–11). Regulatory B cells (Bregs) are particularly important in immune tolerance during TB, modulating CD4+ T cell activity through their expression of programmed death ligand 1 (PD-L1), facilitating the synthesis of granzymes, IL-35, indoleamine 2,3-dioxygenase (IDO) (12–15) and secreting anti-inflammatory cytokines like IL-10 and TGF-β (16, 17). Their role has been extensively studied in autoimmune diseases such as type 1 diabetes mellitus, rheumatoid arthritis, systemic lupus erythematosus, and multiple sclerosis, where anti-inflammatory activity is necessary (18–21). Limited information is available regarding Bregs in the context of TB and helminth co-infection. TB patients exhibit elevated levels of CD19+CD1d+CD5+ Bregs, which suppressed Th17 responses (22). Anti-TB treatment correlated with a decrease in CD19+CD1d+CD5+ Bregs and an increase in the TB antigen-specific IL-22 response (23).

Further studies are needed to elucidate the modulatory role of B cells on T cells during helminth TB co-infection. In this study we conducted an immune profiling of T and B cells in TB patients from a helminth endemic region in Peru, which showed that helminth/TB co-infected individuals have an upregulation of IgM^high^ marginal zone B cells but with similar levels of CD25^+^FOXP3^+^ CD4 Tregs as TB single infected. By using an *in vitro* co-culture model of T and-B cells, in which B cells dampen T cell proliferation and activation (24), we further report that presence of *Ascaris lumbricoides* helminth antigens modulates the IL-15-signaling pathway in T cells and skews B cells to a BCL6^low^ phenotype.

## 2 Materials and method

### 2.1 Ethics statement

For the sample collection of PBMCs from helminth-endemic regions with their controls, all study participants provided written informed consent before their inclusion in the research. Ethical approval for the study protocol was obtained from the Institutional Committee on Research Ethics (CIEI) of Peruvian University Cayetano Heredia.

For the *in vitro* study using heparinized whole blood for PBMCs and as a source of T cells and B cells, the donors for this study were exclusively from Linköping University Hospital blood bank, and all donors were anonymized. This part of the work was entirely conducted in compliance with the Declaration of Helsinki, not requiring a specific ethical approval according to paragraph 4 of the Swedish law.

### 2.2 Study participants

Study participants for the clinical immune profiling were described in our previous work (25). TB patients and individuals from both helminth-endemic regions of Iquitos and Lima, Peru, as well as non-endemic controls from Sweden, were enrolled in the study. The age range of participants was between 18 and 79 years. Participants were categorized into three endemic groups based on their TB and/or helminth infection status: helminth-negative TB-negative healthy endemic controls (Peru control), helminth-negative TB-positive (Helminth-TB+), and helminth-positive TB-positive (Helminth+TB+), in addition to the non-endemic control from Sweden (Sweden control).

This study, is the first of its kind to examine the frequency of T cell and B cell profile in patients with active pulmonary TB and helminth co-infection, and participants were included irrespective of whether they had single or multiple helminth infections. Participants from Lima who were helminth-negative served as endemic controls, while TB patients without helminth infections, as well as those with helminth infections, were predominantly from Iquitos, a region endemic for soil-transmitted helminths (STH) in the Peruvian Amazon. Newly diagnosed TB patients presenting acid-fast bacilli (AFB) smear positivity and clinical symptoms indicative of TB were considered for inclusion in the active pulmonary TB-positive groups. These patients were included in the study if their sputum sample tested positive by the Microscopic Observation Drug Susceptibility (MODS) liquid culture test. For parasitological examination, three stool samples from each participant were screened for helminth infection and species identification using direct microscopy, as well as microscopy after formol-ether concentration using Ritchie’s method and Ziehl-Neelsen staining. Inclusion criteria for TB-negative subjects included having no history of TB disease and presenting none of the classical TB symptoms, such as chronic cough with blood-tinged sputum, fever, or night sweats. Major exclusion criteria for all Peruvian subgroups included individuals who had been on anti-TB drugs for more than one week, recently undergone anti-helminth drug therapy, self-reported as HIV positive, had any underlying or current medical conditions apart from TB or helminth infection (e.g., sepsis, diabetes mellitus, cancer), or were pregnant.

### 2.3 Regulatory T cells and B cells in clinical samples by flow cytometry

PBMCs from Peru were thawed and stained along with PBMC from non-endemic healthy control group (Sweden control). In brief, cells were thawed in RPMI medium contain 10% FBS and centrifuged at 300g for 5 min and resuspended in FACS buffer. Cells were counted and 500,000 cells were stained with antibody cocktails specific for B cell (extracellular staining; CD27 FITC, CD38 PE, IgD PerCP-Cy5,5, CCR9 APC, IgM AF700, CD24 BV421, CD19 BV510) and Treg (CD25 PE, CD45RA AF647, CD3 PO, and CD4 PB as an initial extracellular staining step). All antibodies obtained from Bio Legend. Cells intended for Treg staining were further subjected to fixation and permeabilization using FoxP3 fixation/permeabilization buffer for 30 minutes at 4°C in dark according to the manufacturer’s instructions (BD Biosciences) before intracellular staining with FoxP3 AF488 (BD Biosciences), for 30 minutes. All stained cells were acquired by a Gallios flow cytometer (Beckman Coulter) and analyzed using a Kaluza 2.1 software.

### 2.4 Isolation of T cells and B cells

PBMCs were isolated from heparinized blood obtained from healthy individuals using a standard protocol, in brief whole blood from healthy donor were centrifuged to remove platelet-rich plasma, and then diluted 1:1 with PBS before layering over Ficoll and PBMCs in the interphase collected after centrifugation. T cells and B cells were isolated from the PBMCs by negative selection with the Easy Sep Human T cell or B cell enrichment Kit (STEM cells Technology) in accordance with the manufacture’s instructions. For experiments where T cell proliferation was evaluated, the T cells were labeled with carboxyfluorescein succinimidyl ester (CFSE) fluorescent cell staining dye (5μM; Invitrogen, CA, USA), for 5 minutes, at room temperature (RT).

### 2.5 T cell and B cell co-culture assay

To investigate the regulatory effect of helminth exposure on T cell proliferation and activation we performed a T cell and B cell co-culture *in vitro* assay, where previously B cells have been described to lower T cell proliferation and activation (24). All cells were resuspended in RPMI 1640 containing 10% FBS (Merck), 2mM L-glutamine (Gibco), 200U/ml penicillin, and 100μg/ml streptomycin. For co-culture 4 x 10^4^ T cells and 4 x 10^4^ B cells were cultured in 96 well plates (Eppendorf, Hamburg, Germany), coated with affinipure F(Ab´)2 fragment of goat anti-mouse IgG antibodies (Jackson Immuno research, Cambridgeshire; UK), and activated with soluble anti-CD3 (OKT3; 0.2 μg/ml; Bio Legend, France) and anti-CD28 (CD28; 0.2 μg/ml; Beckman coulter, California, USA) antibodies. Thereafter, CpG (1μM/ml, Invivogen) as positive control for inhibition of T cell proliferation, 5μg/ml *Ascaris lumbricoides* antigen (ASC) obtained from whole worm from Allergen AB Thermo Fisher Scientific, and 5μg/ml *Schistosoma mansoni* soluble egg antigen (SM) from Professor Mike Doenhoff Nottingham University, Nottingham UK, were added. The plates were incubated for 4 days. Experiments were performed in triplicate. On day 4, T cell proliferation was assessed using flow cytometry and co-culture supernatants screened for soluble proteins. Alternatively, day 4 co-cultured cells were removed from the anti-CD3/CD28 environment, washed and rested for 48hours before being stimulated with human recombinant (rh) IL-15, as described below. Concurrently, T cells were cultured in absence of B cells with or without ASC and SM antigens stimulation to evaluate the direct effects of the antigens on T cell proliferation and activation.

### 2.6 Soluble proteins in T and B cell co-culture supernatants

Soluble protein analytes in the supernatants from T and B cell co-culture and T cell cultured in the absence of B cells were quantified using Olink proximity extension immunoassay (PEA) platform with the inflammation panel. This multiplex immunoassay enables the simultaneous measurement of 92 inflammation related human protein biomarkers. The assay was conducted according to the manufacture’s instruction. The resulting Olink-data were normalized to NPX (Normalized Protein eXpression) values on a log2 scale, and significant changes with the presence of helminth antigens presented.

### 2.7 T cell activation and IL-15 receptor expression on day 4 co-cultured cells

Day 4 T cells and B cells in co-culture or T cells cultured in the absence of B cells were gently harvested and centrifuged for 5 min at 900 g RT, and subsequently subjected to flow cytometry staining. Viability assessment by trypan blue indicated above 90% viability, with no differences between the groups. The following antibodies were used for staining: CD215 AF700 (R&D systems), CD122 FITC (Thermofisher scientific), CD132 APC (R&D systems), CD4 BV421 (Biolegend), CD8a APC-fire 750 (Biolegend), CD40L BV510 (Biolegend), CD45RA PE (Biolegend), and CTLA4 PerCP-Cy5.5 (Biolegend). The cells were resuspended and stained in FACS antibody cocktail for 30 minutes, followed by washing with FACS buffer. The cells were acquired using a Gallio’s flow cytometer (Beckman Coulter), and total marker expression (mean fluorescence intensity, MFI) was analyzed using the Kaluza 2.1 analysis software.

### 2.8 rhIL-15 stimulation of rested T and B co-cultures

To assess the effect of recombinant human IL-15 (rhIL-15) on Th1 polarized T cells, T cells cultured in the absence of B cells or T cells and B cells from the day 4 co-culture, described above, were utilized. After four days of co-culture, the cells were gently harvested with culture medium, washed and replated into new wells in presence of 1µg/ml of anti-IL-2. Anti-IL-2 was included to prevent the autocrine IL-2 driven STAT5 activation and to ensure IL-15 specific signaling. The cells were then rested for 48 hours under the same conditions (with or without 5 ug/ml ASC helminth antigen). Following the resting period, the cells were stimulated with 10 ng/ml rhIL-15 (GMH protein, CF (247-GMP, R&D systems)) with newly added anti-IL-2 (1µg/ml). Cells were incubated for 48 or 72 hours before phosphorylation of STAT5 (pSTAT5) and the measurement of transcription factors. Additionally, the Th1 and Th2 profiles were assessed by evaluating the expression of CD183 (CXCR3) for Th1 cells and CD294 (CRTH2) for Th2 cells.

### 2.9 pSTAT5 and transcription factors expression 48 and 72 hours post rhIL-15 stimulation

T cells cultured in the absence of B cells or T cells and B cells, stimulated with rhIL-15 for 48 and 72 hours, were gently harvested, and washed with ice cold PBS supplemented with 0.1% BSA. The cells were utilized for measuring activated STATs (Phosphoflow panel) and transcription factor expression (transcription flow panel). For the phosphoflow panel, cells were stained for surface markers using an antibody cocktail consisting of CD4 BV421 (Biolegend), CD8a APC-Fire 750 (Biolegend), CD19 PE-Cy7 (Biolegend), CD27 AF700 (BD Biosciences), and CD38 BV510 (Biolegend), prepared in PBS with 0.1% BSA. Following a 30-minute incubation cells were washed once and fixed with 4% PFA for 10 minutes at 37°C. The cells were then washed twice with cold PBS containing 1% BSA and permeabilized with 90% methanol for 30 minutes on ice. Following two additional washes with cold PBS containing 1% BSA, the cells were stained intracellularly with an antibody cocktail containing pSTAT1 PerCp-Cy 5.5 (560611, BD biosciences), pSTAT3 PE (612569, BD biosciences), pSTAT5 AF488 (612598, BD biosciences), and pSTAT6 AF647 (612601, BD biosciences) diluted in PBS with 1% BSA for 30 minutes. The cells were washed, resuspended in FACS buffer, and then acquired using the Gallios flow cytometer (Beckman Coulter).

For the transcription flow panel; harvested cells were first stained for surface markers using an antibody cocktail consisting of CD4 BV421 (Biolegend), CD8a APC-Fire750 (Biolegend), CD19PE-Cy7 (Biolegend), CD27AF700 (BD Biosciences), CD38 BV510 (Biolegend), CD183 PerCP-Cy5.5 (Biolegend), and CD294 FITC (Biolegend) diluted in PBS with 0.1% BSA for 30 min at 4°C. Following a single wash, the cells were gently dissociated, then fixed and permeabilized using the FoxP3 Fixation/Permeabilization buffer for 30 min at 4°C according to the manufacturer’s instructions (BD Biosciences). After two washed with 1X permeabilization buffer, the cells were stained at room temperature for 30 min with an antibody cocktail containing BLIMP-1 PE (BD Biosciences) and BCL6 AF647 (BD Biosciences) diluted in 1X permeabilization buffer. Cells were then washed twice with 1X permeabilization buffer, resuspended in FACS buffer, and acquired using a Gallios flow cytometer (Beckman Coulter). Total marker expression measured as MFI, was analyzed using Kaluza 2.1 analysis software.

### 2.10 Statistics

All quantitative data are presented as mean ± standard error of the means (SEM). For comparison among multiple groups, a one-way repeated measures ANOVA was used, followed by Dunnett’s or Holm-Sidak’s multiple comparison test. Differences were considered statistically significant at p < 0.05. Statistical analyses were performed using GraphPad Prism version 9 (GraphPad Software, USA).

## 3 Results

### 3.1 Increased frequencies of regulatory B lymphocytes and T lymphocytes in helminth/TB co-infected individuals

**Figure 1:**
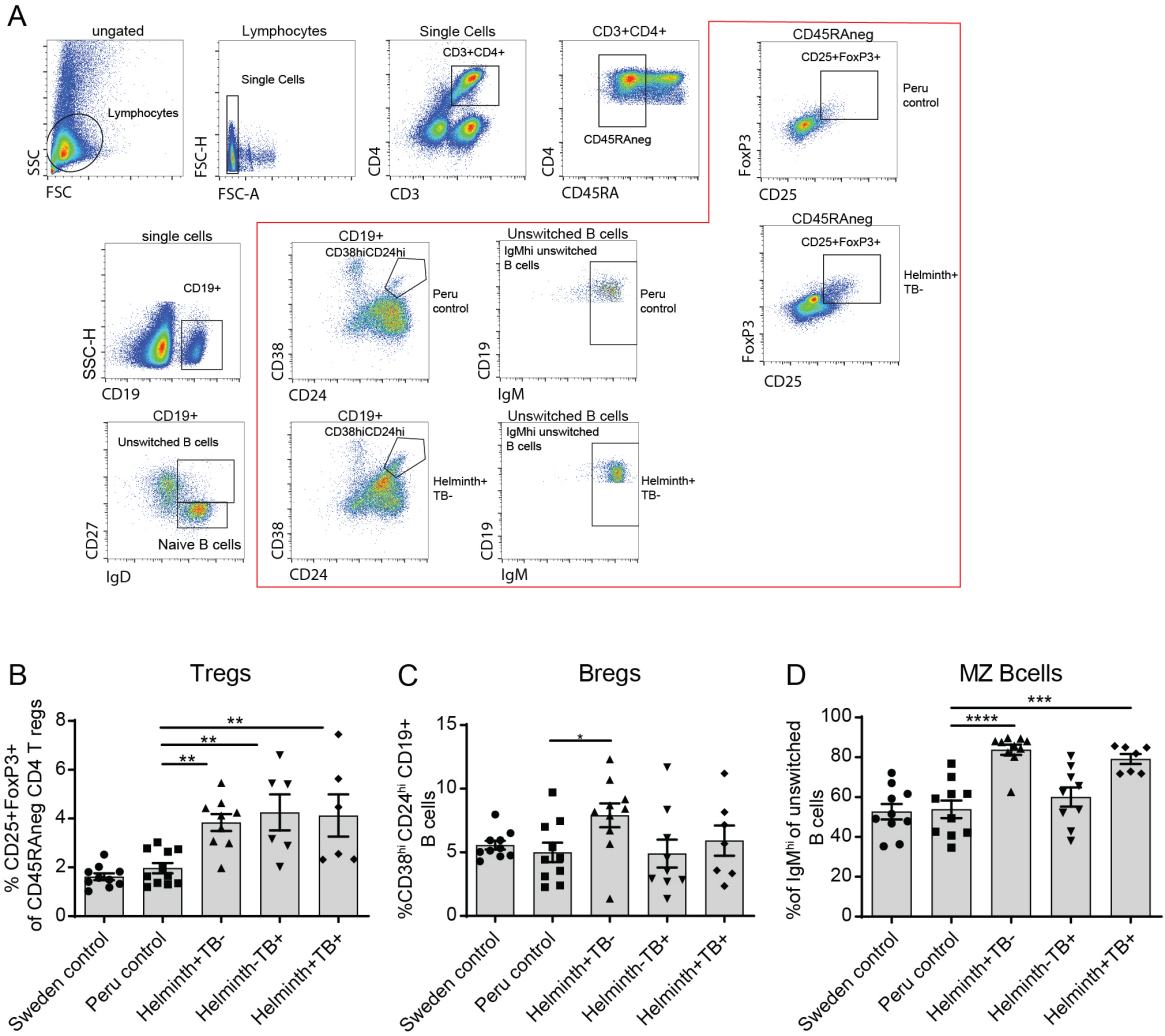
Increased frequency of MZ B cells in helminth co-infected TB patients. PBMCs from healthy individuals (Peru control, n=10-11), helminth infected (Helminth+TB-, n=9-10), TB patients helminth negative (Helminth-TB+, n=6-9), and TB patients with helminth (Helminth+TB+, n=6-8) from a Peruvian cohort, and using a Swedish non-endemic healthy control group (Sweden control, n=10) as reference, were evaluated by flow cytometry. Flow cytometry gating for Tregs, Breg cells, and MZ B cells, with representative donors highlighted within red box **(A)**. Frequency of CD25^+^FoxP3^+^ Tregs of memory T cells **(B)**, CD38^+^CD24^+^CD19^+^ Bregs **(C)**, and IgM^high^ CD27^+^IgD^+^CD19^+^ unswitched B cells (MZ B cells) **(D)** are shown. Scatter plot overlaid on bar graph showing individual data points and bars representing mean ± SEM with *, p<0.05; **, p<0.01; ***, p<0.001; ****, p<0.0001 using one-way ANOVA with Dunnett’s multiple comparison test.

To investigate the frequencies of regulatory cells and how their expression changes during helminth co-infection, we performed immune profiling of T and B lymphocytes in a Peruvian cohort. The study population consisted of a Peruvian control group (n=11), a helminth-infected (no TB) group (n=11), a TB-infected (no helminth) group (n=8), a helminth/TB co-infected group (n=8), and a Swedish non-endemic control group (n=10). Analysis of the frequency of CD25^+^FoxP3^+^ Tregs showed a significantly elevated frequency of Tregs in the helminth-infected, TB-infected, and helminth/TB co-infected groups compared to the endemic Peruvian control (**Figure 1B**), with frequencies of CD4^+^ T cell being the same in all groups (**Supplementary Figure S1**). Conversely, the frequency of CD19^+^CD24^+^CD38^+^ Bregs was significantly elevated only in the helminth-infected group (**Figure 1C****).** Additionally, we observed that a specialized subset of B cells, CD19^+^CD27^+^IgD^+^IgM^high^ MZ B cells, exhibited a significantly increased frequency in both the helminth/TB co-infected and the helminth-infected groups, but not in the TB-infected group (**Figure 1D**). Collectively, this indicates that the helminth-infected and helminth/TB co-infected groups showed an increased frequency of regulatory cells.

### 3.2 B-cell-regulated T cell proliferation was not modulated by helminth exposure

**Figure 2:**
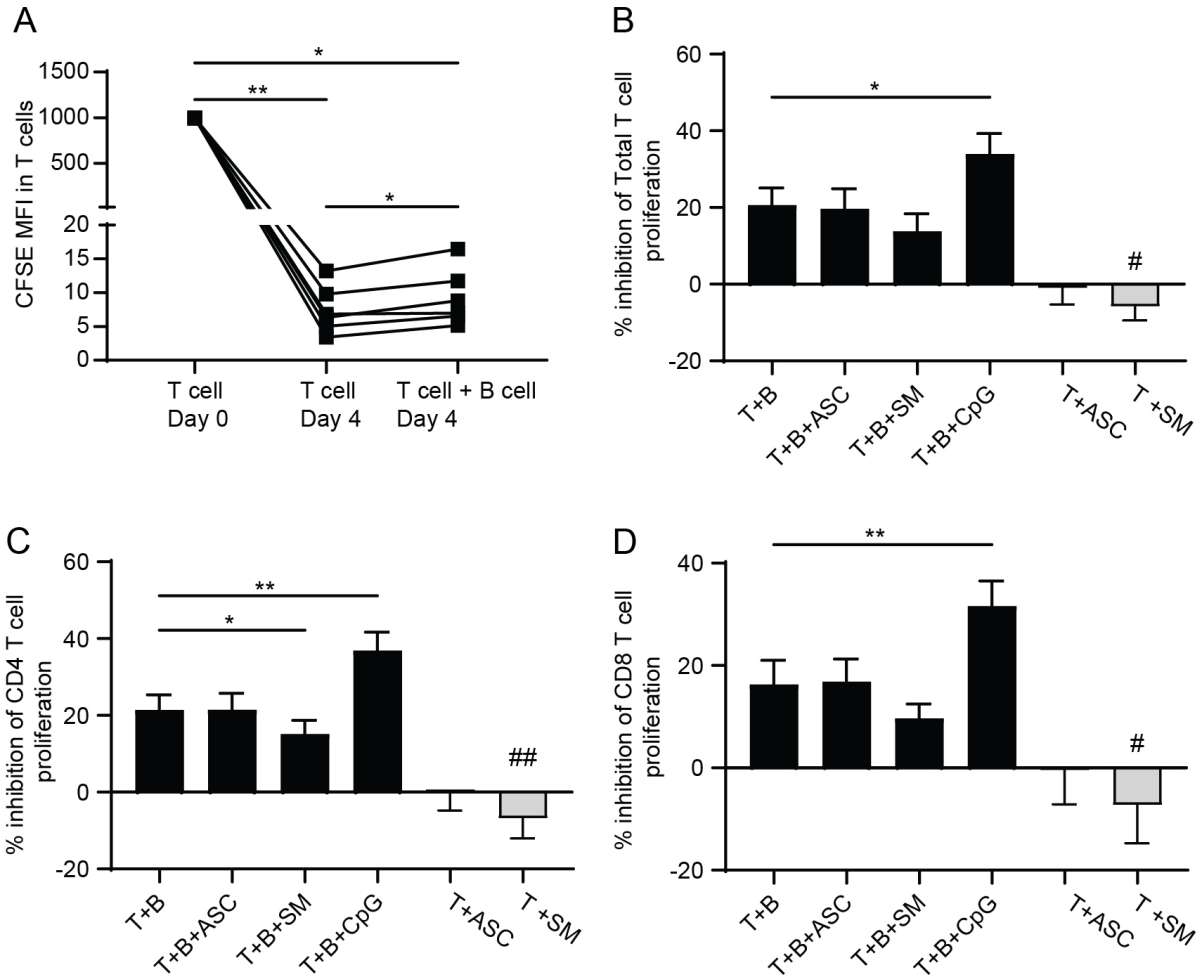
T cell proliferation is inhibited by the presence of B cells and not affected by helminth antigen exposure. Day 0 and Day 4 T cell proliferation in activated T cells in presence and absence of B cells measured using CFSE **(A)**. Percent inhibition in T cell proliferation with or without B cells, analyzed as percent inhibition in all CD3+, total T cells **(B)**, in CD4 T cells **(C)**, and in CD8 T cell **(D)**, at 4 days B cell T cell co-culture without or with *Ascaris lumbricoides* protein antigen exposure (ASC), *Schistosoma mansoni* soluble egg antigen (SM), or CpG. Lines indicate the individual donor data expressed as mean ± SEM with #*, p<0.05 and **, p<0.01, n=6 independent PBMC donors, using one-way ANOVA with Dunnett’s multiple comparison test.

As our clinical observations indicated an increased frequency of Bregs with helminth and helminth/TB co-infection, we performed an *in vitro* assay for T cell proliferation in co-culture with autologous B cells and presence or absence of helminth antigen exposure, with the working hypothesis that the presence of helminth antigens would further increase the inhibitory action of B cells on T cell proliferation (24). Using the CFSE-dilution assay, T cell proliferation was assayed at day 0 and day 4 post co-culture with B cells. At day 0, T cells exhibited a high MFI of CFSE, and as the cells proliferated the CFSE signal was reduced and used as a measure of proliferation. By day 4, there was an increased T cell proliferation, which was significantly decreased by the presence of B cells (**Figure 2A**), as previously shown (24). Further evaluation of the percentage difference in the inhibition of T cell proliferation by B cells, was conducted based on the proliferation rate of T cells cultured alone. Four days post-co-culture, a significantly higher inhibition of T cell proliferation was recorded with the positive control CpG, compared to T cells and B cells alone (**Figure 2B**), whereas helminth exposure using ASC did not affect T cell proliferation. Exposure to SM, significantly reduced the inhibitory capacity of B cells in CD4+ T cells, but not in CD8+ T cells of the co-culture, when compared to T and B cells without helminth antigen exposure (**Figures 2B–D**; black bars). However, in the absence of B cells, Schistosoma antigen exposure (SM) led to a significantly reduced inhibition of T cell proliferation both in total T cells and in the sub-analysis of CD4+ and CD8+ T cells (**Figures 2B–D**; gray bars), suggesting that the modulatory effect of this antigen on T cell proliferation is more pronounced in absence of B cell-mediated regulation.

### 3.3 Increased levels of IL-15Rα and decreased IL-2/15Rβ in T cell B cell co-cultures exposed to ASC

To further examine T cell activation and the resulting inflammatory mediators generated in the T cell B cell co-culture, or T cell cultured in the absence of B cells, the supernatant from the *in vitro* co-culture was screened to identify proteins associated with inflammation and immune regulation using the inflammation panel by Olink. Compared to T and B co-cultures without helminth exposure, ASC helminth antigen exposure exhibited elevated levels of interleukin-15-receptor subunit alpha (IL-15Rα), and C motif chemokine 19 (CCL19), along with significantly elevated levels of TNFβ (Lymphotoxin-alpha), interleukin-10-receptor subunit alpha (IL-10RA). In contrast, the levels of interleukin-2/15-receptor subunit beta (IL-2/15Rβ), IL-24, and C-X-C motif chemokine 11 (CXCL11) were significantly decreased. SM helminth antigen exposure showed increased levels of CCL19 along with significantly increased levels of C-X-C motif chemokine 9 (CXCL9), C-C motif chemokine 4 (CCL4), interferon gamma (IFNγ), and TNFβ. Conversely, levels of LAP TGFβ-1, T-cell surface glycoprotein CD5 (CD5), tumor necrosis factor receptor superfamily member 9 (TNFRSF9) and T cell surface glycoprotein CD8 alpha chain (CD8A) were significantly decreased with SM (**Table 1**).

**Table 1:** Proteins modulated in T cells and B cells co-culture system following exposure to helminth antigens.

Proteins upregulated/downregulated with *Ascaris lumbricoides* or *Schistosoma mansoni* antigen exposure in T+B co-culture			
Protein	T+B (mean± SEM)	T+B+ ASC (mean± SEM)	T+B+SM (mean± SEM)
IL-15RA	0,27 ± 0,08	0,67 ± 0,29	0,18 ± 0,09
TNFB	11,53 ± 0,01	11,62 ± 0,03*	11,63 ± 0,01**
IL-10RA	1,27 ± 0,07	1,67 ± 0,14*	1,18 ± 0,08
CCL19	4,671 ± 0,35	5,47 ± 0,62	5,31 ± 0,30
IFN-γ	18,03 ± 0,20	18,16 ± 0,24	18,30 ± 0,15*
TSLP	0,75 ± 0,23	0,93 ± 0,26	1,19 ± 0,25**
CCL4	11,63 ± 0,07	11,69 ± 0,12	11,83 ± 0,06*
CXCL9	9,23 ± 0,08	9,31 ± 0,04	9,40 ± 0,08*
IL-2RB	0,74 ± 0,18	0,00 ± 0,00**	0,90 ± 0,07
IL-24	6,17 ± 0,12	5,99 ± 0,13*	6,04 ± 0,08
LAP TGF β-1	3,81 ± 0,20	3,74 ± 0,20	3,61 ± 0,23 **
CXCL11	6,04 ± 0,13	5,72 ± 0,05*	5,85 ± 0,10
CD5	9,25 ± 0,10	9,12 ± 0,09	9,02 ± 0,15*
TNFRSF9	12,42 ± 0,11	12,39 ± 0,08	12,20 ± 0,11*
CD8A	10,21 ± 0,14	9,94 ± 0,11	9,89 ± 0,08*
TGFα	2,33 ± 0,17	2,24 ± 0,18	2,22 ± 0,16
Proteins upregulated/downregulated with *Ascaris lumbricoides* or *Schistosoma mansoni* antigen exposure in T cell cultured the in absence of B cell			
Protein	T (mean± SEM)	T+ ASC (mean± SEM)	T+SM (mean± SEM)
TSLP	0,53 ± 0,32	0,91 ± 0,13	1,47 ± 0,35*
IL-10RA	1,12 ± 0,15	1,53 ± 0,05*	1,28 ± 0,11
CD40	7,36 ± 0,40	7,68 ± 0,34*	7,56 ± 0,40
CSF-1	11,25 ± 0,13	11,47 ± 0,07	11,36 ± 0,11
CASP-8	5,59 ± 0,26	6,49 ± 0,31**	6,49 ± 0,35**
IL-2RB	0,57 ± 0,21	0,11 ± 0,20	1,06 ± 0,22

CD3/CD28a activated T cells were cultured alone (T) or with autologous B cells (T+B) and stimulated with *Ascaris lumbricoides* (ASC) or *Schistosoma mansoni* (SM) antigens. Protein levels were measured in culture medium after 4 days by Olink and results expressed as mean ± SEM of NPX values (NPX, Log_2_ scale). Statistical significance compared to T cells or T+B co-cultures without antigen stimulation was assessed using one-way repeated measures ANOVA with Dunnett’s multiple comparisons test, with p < 0.05 (*) and p < 0.01 (**), n = 5 donors.

For T cells cultured in the absence of B cells, ASC helminth antigen exposure induced significantly elevated levels of IL-10RA, CD40, Macrophage colony-stimulating factor-1 (CSF-1), and Caspase-8 (CASP-8). Whereas SM helminth antigen exposure induced significantly elevated levels of thymic stromal lymphopoietin (TSLP) and CASP-8. Interleukin-2/15-receptor subunit beta (IL-2/15Rβ) was found to decrease in exposure with ASC helminth antigen (**Table 1**).

A unique observation was the increased levels of IL-15Rα and decreased IL-2/15Rβ protein following ASC exposure in the T and B cells co-culture system. IL-15Rα is the high affinity binding receptor, specific for IL-15 and involved in trans-presentation of IL-15 facilitating the activation of cells expressing IL15Rβ and γc (26). IL-15 is involved in the growth and stimulation of activated T cells, in the induction of cytolytic effector cells, and in B cell co-stimulation for proliferation and immunoglobulin production (27).

### 3.4 ASC helminth antigen exposure resulted in increased expression of IL-15Rα (CD215) and IL-2/15Rβ (CD122) on T cells

**Figure 3:**
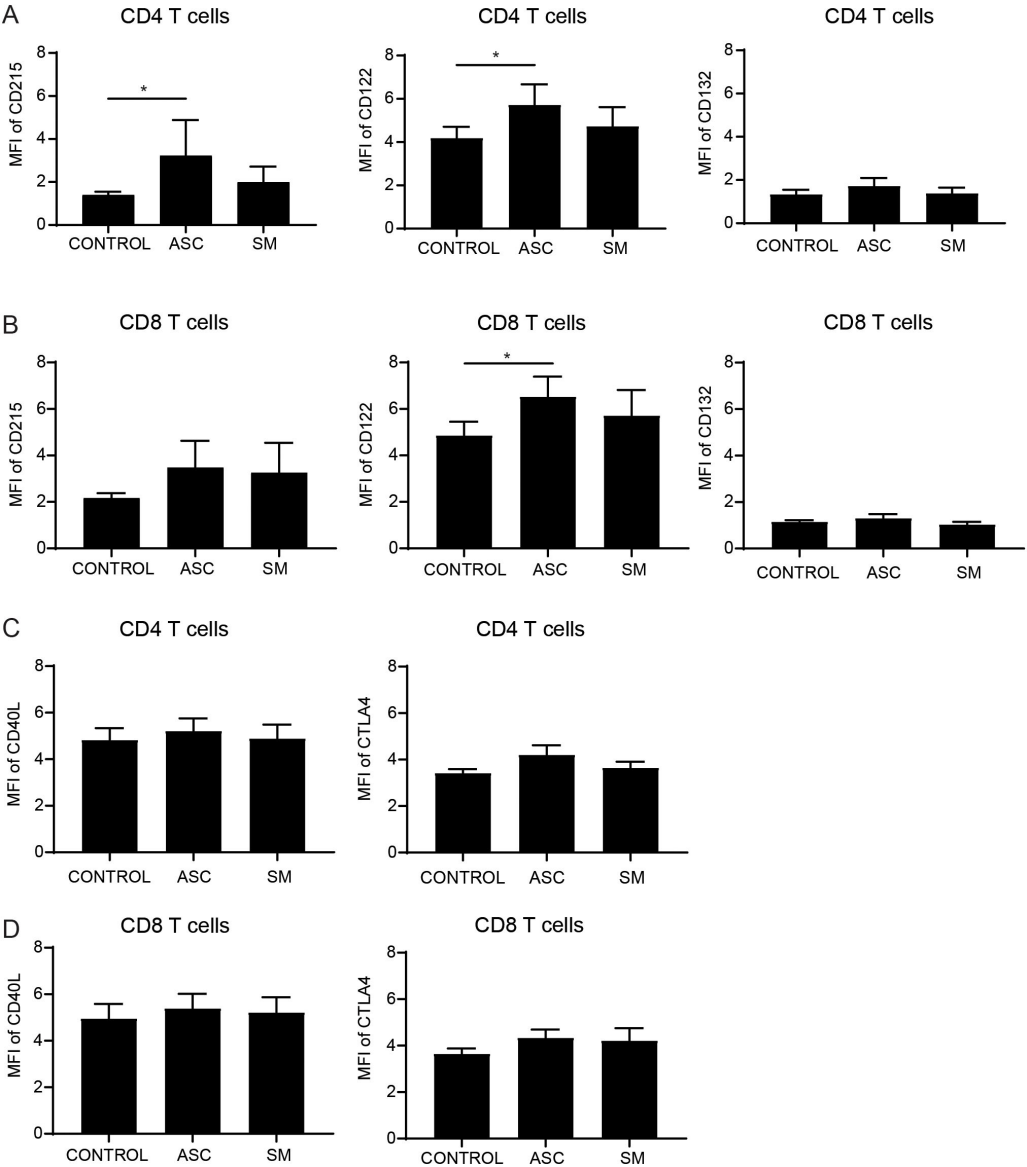
Increased IL-15 receptor expression on CD4 and CD8 T cells with ASC exposure of T and B cell co-cultures. Expression of IL-15Rα (CD215), IL-2/15Rβ (CD122), IL-2/15Rγ (CD132) on CD4 T cells **(A)** or CD8 T cells **(B)**, and CD40L and CTLA4 on CD4 **(C)** or CD8 **(D)** T cells measured after day 4 co-culture with B cell and activated T cells in presence/absence of helminth antigen exposure. Data expressed as mean ± SEM with *, p<0.05, n=6, using one-way ANOVA with Dunnett’s multiple comparison test.

As there was an increased level of soluble IL-15Rα in the co-culture supernatant when exposed with ASC helminth antigen, the surface expression of IL-15Rα (CD215), IL-2/15Rβ (CD122) and IL-15 receptor common gamma chain (IL-2/15Rγ; CD132) on activated T cells, and B cells, four days after co-culture were determined. Surface expression analysis by flow cytometry showed that IL-15Rα and IL-2/15Rβ was significantly increased on CD4 T cells exposed with ASC helminth antigen (**Figure 3A**), whereas for CD8 T cells there was a non-significant increase of IL-15Rα and a significant increase of IL-2/15Rβ (**Figure 3B**). This pattern of IL-15 receptor expression on T cells was not observed with SM helminth exposure, and neither helminth exposure affected the B cells expression of these receptors (**Supplementary Figure S2**). Taken together, ASC exposure led to elevated levels of soluble IL-15Rα in the supernatant, with a concomitant upregulation of surface IL-15Rα expression on CD4 T cells, suggesting that the rise in soluble IL-15Rα is not due to loss of membrane receptor alone but more likely reflects enhanced IL-15Rα production with concurrent release of soluble forms. Additionally, the measured activation and inhibitory marker expression on T cells on day four, showed no significant differences for the activation marker CD40L and the inhibitory marker CTLA4 on CD4 (**Figure 3C**) and CD8 T cells (**Figure 3D**).

### 3.5 rhIL-15-induced activation of STAT5 is downregulated by ASC helminth antigen exposure

**Figure 4:**
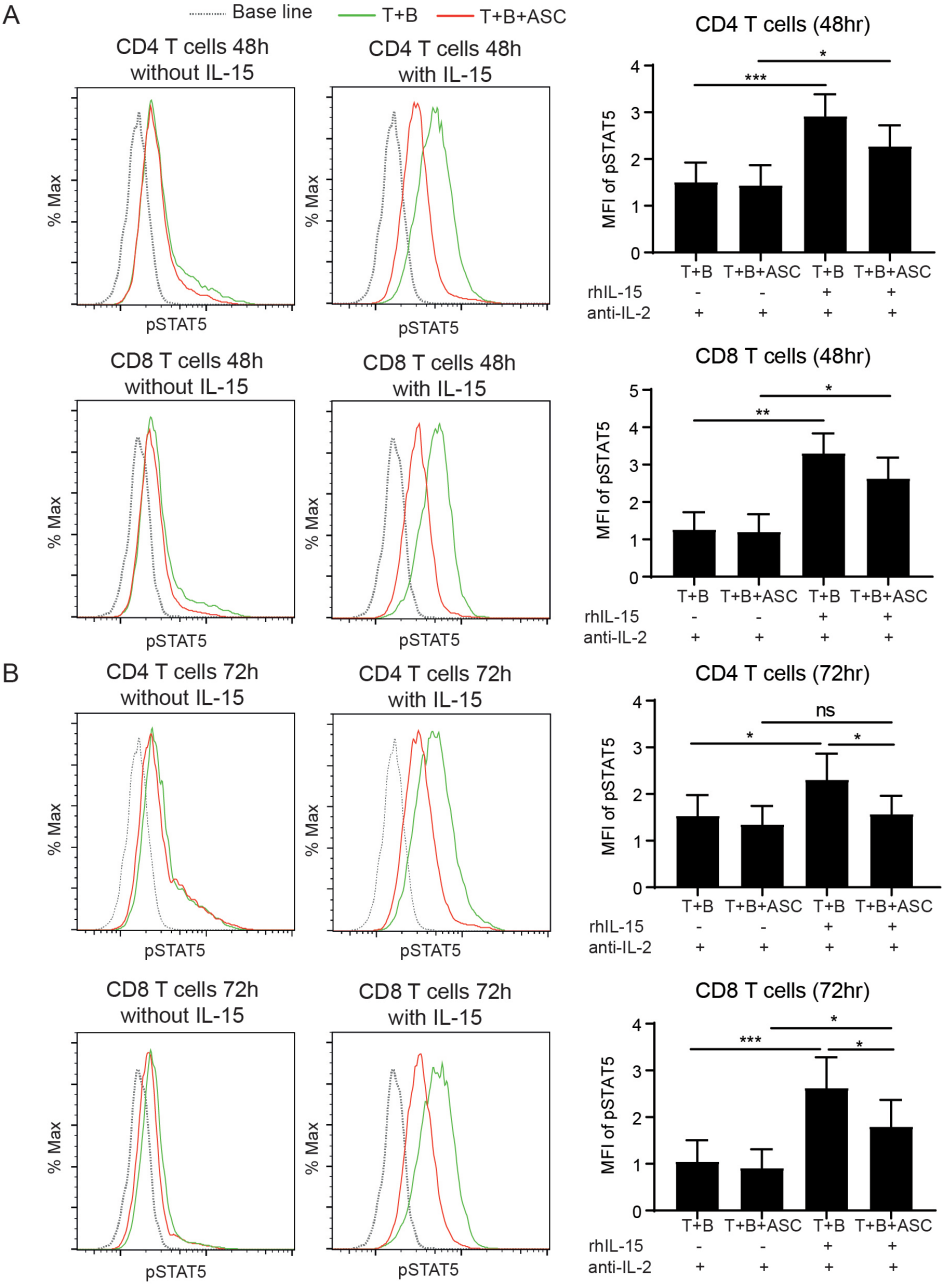
rhIL-15-induced STAT5 phosphorylation is decreased in activated T cells of co-cultures exposed to ASC. Phosphorylated STAT5 (pSTAT5) measured using intracellular staining and flow cytometry. pSTAT5 after 48 hours of rhIL-15 stimulation in CD4 T cell and CD8 T cells **(A)**, and 72 hours post rhIL-15 stimulation **(B)**. To the right, data expressed as mean ± SEM with *, p<0.05; **, p<0.01; ***, p<0.001, for n=6 individual donors, using one-way ANOVA with Holmsidak comparison test. Representative flow cytometry histograms for pSTAT5 signal intensity are shown to the left.

The upregulated expression of IL-15Rα and IL-15Rβ on T lymphocytes with ASC exposure prompted us to further assess the responsiveness of these cells to IL-15 stimulation. Using a STRING-based protein–protein interaction analysis of proteins found modulated in supernatants of co-cultures exposed to ASC, additionally identified enrichment of IL-15 receptor signaling components (**Supplementary Figure S3**), suggesting modulation of IL-15 pathway in response to helminth antigen exposure. Given the established role of IL-15 in activating the JAK–STAT signaling cascade, particularly STAT5, we evaluated the phosphorylation of STAT5 in activated T cells at 48- and 72-hours following stimulation with rhIL-15. To specifically assess IL-15-driven STAT5 phosphorylation, endogenous IL-2 signaling was neutralized using anti-IL-2 antibodies during the resting phase and the rhIL-15 restimulation phase. The 48- and 72-hour time-points for stimulation with rhIL-15 were chosen based on previous findings indicating that T cells stimulated with rhIL-15 *in vitro* have a sustained phosphorylation of STAT5 for 72h (due to recirculation of IL-15/IL-15Rα complex maintaining the signal) (28). Our findings indicate that rhIL-15 induces increased phosphorylation of STAT5 in both CD4+ and CD8+ T cells at 48 h. ASC exposure in co-cultures attenuated this effect, although STAT5 phosphorylation remained significantly higher than in absence of rhIL-15 (**Figure 4A**). At 72 h post rhIL-15 stimulation of co-cultures, T cells in the control group maintained significantly increased STAT5 phosphorylation in both CD4+ and CD8+ T cells, whereas ASC exposure in CD4+ T cells resulted in reduced STAT5 phosphorylation to levels of that in samples without rhIL-15 stimulation. STAT5 phosphorylation in ASC exposed CD8+ T cells and rhIL-15-stimulation, showed significantly lower levels of STAT5 phosphorylation compared to control with rhIL-15, yet still maintained significantly higher levels than that in ASC without rhIL-15 (**Figure 4B**). Analysis of surface markers for Th1 (CD183) and Th2 (CD294) before and 72h after rhIL-15 stimulation, showed that all T cells expressed a high and maintained CD183 level with no expression of CD294 (**Supplementary Figure S4**). There was no increased phosphorylation of pSTAT1 (Th1), pSTAT3 (Th17), or pSTAT6 (Th2) in T cells in response to rhIL-15 at 48h post-stimulation. At 72h post IL-15 stimulation, however, there was a significant increase in STAT1 phosphorylation in CD8+ T cells in the control group indicating increased Th1 activation, which was not apparent in the T and B co-cultures exposed to ASC (**Supplementary Figure S5**). Although phosphorylated STAT1 levels in CD4+ T cells were not increased in the control group following rhIL-15 stimulation at this timepoint, ASC exposure under the same stimulation led to a significant decrease in STAT1 phosphorylation. Furthermore, rhIL-15 stimulation decreased STAT3 and STAT6 phosphorylation in CD4+ T cells only in the control group. These findings indicate that ASC exposure impairs T cells responsiveness to IL-15, resulting in either an induced (CD8+ T cells) or maintained (CD4+ T cells) Th1 activation defect.

Further we observed the impact of ASC helminth antigen exposure on IL-15 stimulated T cells cultured in the absence of B cells at the 72 h time point (**Supplementary Figure S6**). In CD4^+^ T cells, rhIL-15 stimulation led to reduced STAT1 phosphorylation, that was further decreased upon ASC exposure. Similarly, STAT3 phosphorylation was significantly reduced in ASC-exposed, rhIL-15-stimulated cells. In CD8^+^ T cells, ASC exposure alone significantly reduced STAT1 phosphorylation, which was restored by rhIL-15 stimulation. Regarding STAT5 phosphorylation, rhIL-15 stimulation significantly increased STAT5 phosphorylation in CD8+ T cells from control cultures, whereas STAT5 phosphorylation was not significantly altered in CD4 T cells.

### 3.6 rhIL-15 exposure represses the transcription factor BCL6 in B cells during ASC antigen exposure

**Figure 5:**
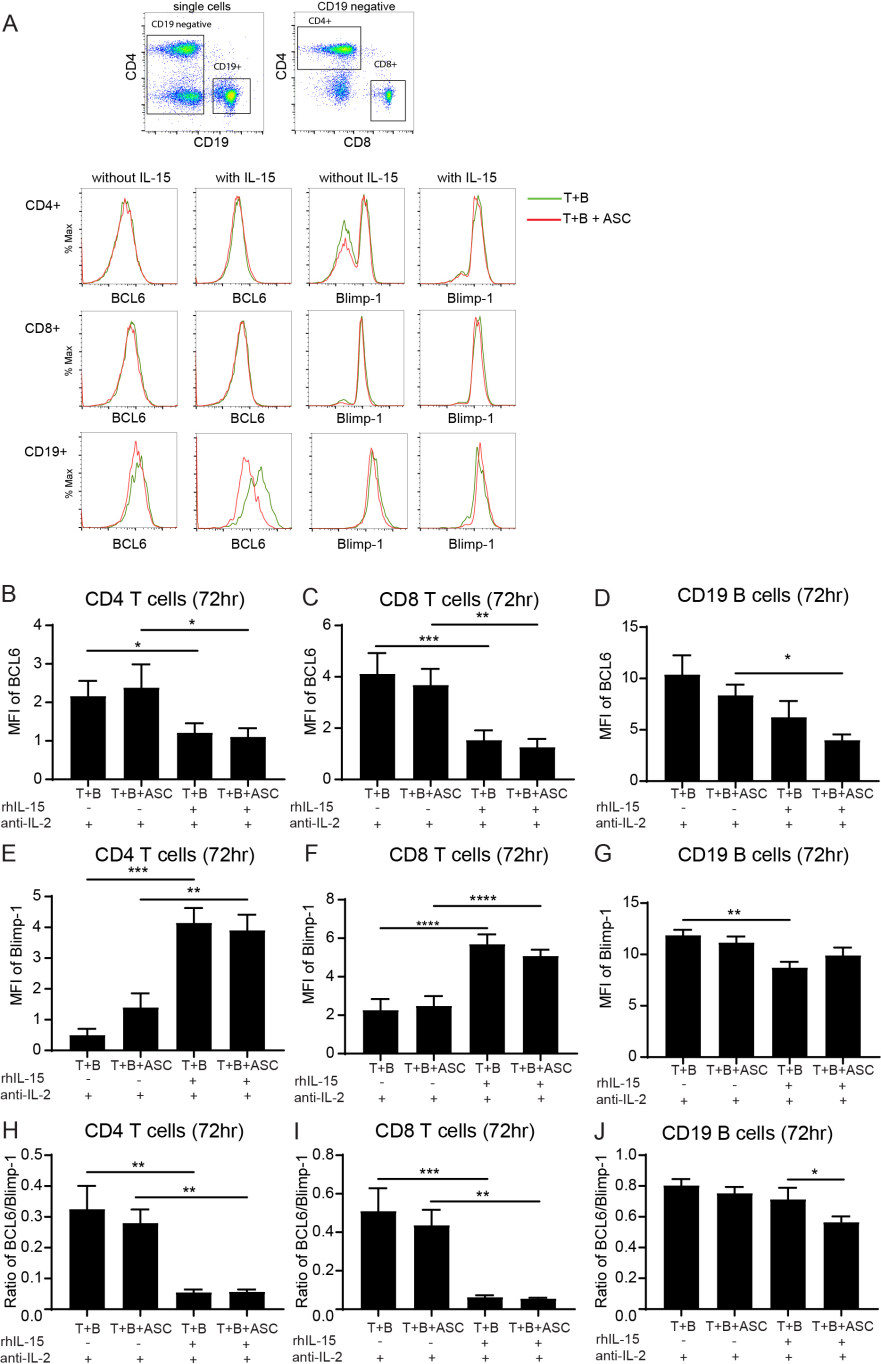
Decreased BCL6 in B cells of ASC exposed co-cultures with activated T cells stimulated with rhIL-15. BCL6 and BLIMP-1 transcription factor expression in T cell B cell co-cultures measured using flow cytometry 72 hours post rhIL-15 stimulation. Flow cytometry gating of CD4+, CD8+, and CD19+ lymphocytes with representative donor expression of BCL6 and BLIMP-1 **(A)**. Mean fluorescence intensity (MFI) of transcription factor BCL6 in CD4 T cell **(B)** CD8 T cells **(C)** and B cells **(D)**. BLIMP-1 expression in CD4 T cell **(E)**, CD8 T cells **(F)** and B cells **(G)**. BCL6/BLIMP-1 ratio CD4 T cell **(H)**, CD8 T cells **(I)** and B cells **(J)**. Data expressed as mean ± SEM with *, p<0.05; **, p<0.01; ***, p<0.001; ****, p<0.0001, n=6, using one-way ANOVA and paired comparisons for ratios using lognormal assumption and paired t-test (geometric mean).

To evaluate the expression of transcriptional regulators B-lymphocyte-induced maturation protein 1 (BLIMP-1) and B-cell lymphoma 6 (BCL6) in activated T cells and B cells under rhIL-15 stimulation, we measure expression of BLIMP-1 and BCL6 in T cells and B cells after 72 hours of rhIL-15 exposure. Representative flowcytometry gating and donor histograms are shown in (**Figure 5A****).** Our results show that 72 hours of rhIL-15 stimulation resulted in a significant decrease in BCL6 expression in CD4+ T cells (**Figure 5B**) and CD8+ T cells (**Figure 5C**), accompanied by a significant upregulation of BLIMP-1 in CD4+ T cells (**Figure 5E**) and CD8+ T cells (**Figure 5F**). This enhanced expression of BLIMP-1 and suppression of BCL6 was similarly observed with ASC helminth antigen exposure. The BCL6/BLIMP-1 ratio was substantially and significantly reduced after 72 hours of rhIL-15 stimulation, irrespective of helminth exposure (**Figures 5H, I**). In B cells from the same co-cultures, there was a significant decrease in BCL6 expression after stimulation with rhIL-15 in the ASC exposed group, compared to ASC exposed without rhIL-15 (**Figure 5D**). On the other hand, controls had a significant decrease in BLIMP-1 with rhIL-15, compared to without rhIL-15 (**Figure 5G****).** Moreover, the BCL6/BLIMP-1 ratio was significantly decreased after rhIL-15 stimulation in the ASC helminth antigen exposed group, compared to the control without ASC exposure (**Figure 5J**).

## 4 Discussion

Several studies on immune modulation during helminth and mycobacterial co-infection indicate that helminth appears to dampen the proinflammatory cytokine response while promoting a regulatory- or Th2-mediated immune response. In this study, we investigated the helminth associated modulation of T cells and B cells in individuals from Peru, and evaluated the impact of helminth antigens on B-cell-regulated T cell activation and proliferation *in vitro*. For this we used a T cell B cell co-culture system where B cells dampen T cell activation (24). Our clinical data revealed a significant increased frequency of CD25^+^Foxp3^+^ Tregs in all infected Peruvian individuals. Notably, a significant increase in the frequency of MZ B cells was observed exclusively in individuals with helminth single-infection and helminth/TB co-infection. *In vitro* co-culture experiments demonstrated that B cells inhibited T cell proliferation and although ASC had no effect, SM partly reduced the B cell inhibited T cell proliferation. The presence of ASC helminth antigen, however, resulted in significantly elevated levels of soluble IL-15Rα in the co-culture supernatants with a concurrent increase in surface IL-15Rα and IL-2/15Rβ on CD4+T cells. rhIL-15 stimulation experiments of these co-cultures, for elucidating possible modulation by ASC, showed an increased STAT5 phosphorylation that was reduced with ASC exposure in CD4+ T cells by the 72h post-rhIL-15 timepoint. The long-term effect (72h) of ASC on rhIL-15 stimulation also indicated an inability of T cells to respond to IL-15 with an induced (CD8+ T cells) or maintained (CD4+ T cells) Th1 activation. Although transcription factors analysis of rhIL-15 stimulated co-cultures at this timepoint showed an increased BLIMP-1 and decreased BCL6 expression in CD4+ T cells and CD8+ T cells regardless of ASC exposure, B cells exhibited significantly decreased BCL6 expression with ASC exposure that may reflect a switch to a more BCL6^low^ and differentiated B cell, such as MZ B cells (29).

The role of Tregs in modulating immune responses is evident in conditions requiring inflammation attenuation, though their role may be counterproductive in pathogen containment. Increased Tregs frequency indicates effective control of excessive immune response in conditions such as rheumatoid arthritis, multiple sclerosis, systemic lupus erythematosus, and during infection with helminth, HIV, and TB (30–35). Thus our data on increased Tregs frequency in helminth, TB and helminth/TB co-infected individuals may indicate the amelioration of inflammation caused by different pathogens. Notably, increased Tregs levels have been reported during infections with *Heligmosomoides polygyrus*, *Litomosoides sigmodontis*, *Schistosoma japonicum* and *Strongyloides ratti*, where Tregs experimentally also was shown to be important for parasite survival (36).

B cells have previously been shown to induce immune regulation through the initiation of Bregs and the secretion of regulatory cytokines thereby directly interacting with T cells (37–39). Breg produced IL-10 and TGF-β suppress CD4 T cells and mediate the induction of Tregs (40). Additionally, Bregs have been found to reverse allergen-induced airway inflammation by inducing Tregs (41), indicating their beneficial role in inflammatory conditions. Similarly, TB is known to cause lung inflammation by induction of cytokines, such as IFN-γ, IL-1β, IL-2, IL-12 and TNF (42). Decreased frequency of Bregs was associated with severe COVID-19, with the reduction in Bregs linked with the development of septic shock (43, 44). Our findings demonstrates an increased frequency of MZ B cell in individuals with helminth single-infection and those with helminth/TB co-infection. MZ B cells have been shown to increase in active TB cases (45), which we do not see in this cohort of TB patients were stool sample analysis for helminth infection is performed. In response to Mtb infection MZ B cells accumulate in the lungs and spleen and exhibit a memory-like phenotype producing multiple cytokines and restricting infection by coordinating cell-mediated immunity (46).

Similar to our T and B cell co-culture experiments, others also report that B cells reduce the proliferation of activated T cells (22, 24). We show that ASC helminth antigen exposure did not interfere with the B cell inhibition of T cell proliferation. However, we show the novel finding of attenuated IL-15 signaling in CD4 and CD8 T cells during ASC helminth antigen exposure. ASC-exposed T and B cell co-cultures showed an increase in soluble IL-15Rα together with increased expression of IL-15Rα and its coreceptor IL-2/15Rβ on CD4 T cells and CD8 T cell (only IL-2/15Rβ for CD8 T cells). It is known that IL-15α shedding is involved in IL-15 presentation to responding cells (47), through the trans presentation mode by which the IL-15/IL-15Rα complex is presented to IL-2/15Rβγ on the cell surface, after which the IL-15/IL-15Rα complex is internalized by effectors cells like NK, B and T cells (48), prolonging cell survival (26). This complex initiates the activation of Janus kinase1 and Janus kinase 3 which further induce activation of IL-15 regulated genes via STAT3 and STAT5 (49). Aberrant levels of IL-15 have been associated with numerous inflammatory diseases such as Crohn’s disease, rheumatoid arthritis, and ulcerative colitis.

Our research is conducted in the context of tuberculosis, applying a Th1-induced activation of T cells by aCD3/aCD28. In our study, ASC exposure resulted in markedly reduced and transient STAT5 phosphorylation in CD4^+^ T cells following rhIL-15 stimulation. STAT5 activation downstream of IL-15 signaling plays a critical role in T cell survival, proliferation, metabolic fitness, and Th1 effector differentiation, while also repressing TFH differentiation through antagonism of BCL6 (28). Attenuation of IL-15-STAT5-signaling during ASC exposure therefore suggests a potential impairment of Th1 responses, with possible consequences for anti-mycobacterial immunity. However, excessive IL-15 signaling also poses challenges, as IL-15–driven inflammation can contribute to significant comorbidity. Indeed, increased IL-15 levels in bronchial biopsy specimens from tuberculosis patients have been associated with inflammatory lesions in the lung parenchyma (50). Our data suggest that ASC helminth exposure might modulate inflammation by interrupting the IL-15 activation pathway. The critical role of IL-15 in the immune response associated with inflammatory disease was previously demonstrated (51). Notably, it was shown that IL-15Rα can be cleaved by a metalloproteinase-dependent proteolytic mechanism generating an antagonistic soluble IL-15Rα that retains a high affinity for IL-15 and capable of inhibiting biological activity of IL-15 at very low concentration (52). Although soluble IL-15Rα is often generated by proteolytic shedding, IL-15Rα expression is also regulated at the transcriptional level, and alternative splicing can yield secreted isoforms (52). The simultaneous increase in soluble and surface IL-15Rα observed following ASC exposure supports a model in which ASC induces upregulation of IL-15Rα, potentially accompanied by shedding or secretion of soluble variants, rather than shedding as the sole mechanism. These insights suggest a potential mechanism by which ASC helminth exposure could mitigate excessive inflammation, offering a novel perspective on the modulation of immune responses in the context of tuberculosis. Further research is needed to validate these findings and fully understand the implications of ASC helminth exposure on IL-15 signaling pathways and T cell functionality.

Our data, showing elevated levels of IL-15Rα, without indicating the presence of IL-15 (data not shown), suggests that our system does not contain cells capable of secreting IL-15 (53). This prompted us to utilize rhIL-15 to facilitate the IL-15 pathway. As previously illustrated IL-15 exposure with its high affinity receptor IL-15Rα leads to sustained phosphorylation of STAT5 up to 72 hours (28). In accordance with these findings, we observed that IL-15 stimulation maintained STAT5 phosphorylation at 48 and 72 hours. Prior to assessing pSTAT5, we utilized anti-IL-2 to exclude IL-2 induced polarization and activation of Th1 cells (54, 55), added after replating and resting the cells. Another reason for removing the cells from the aCD3/aCD28 environment and resting them is that even a low concentrations of aCD3/aCD28 can induce pSTAT5 (56). Collectively, our data show that ASC helminth antigen exposure, followed by IL-15 stimulation induces a limited STAT5 phosphorylation that does not sustain for 72 hours. STAT5 plays an important role in maintaining Th1 proliferation and activation of specific T cell subsets such as Th1, Th2, and Treg cells, inhibiting the development of T_FH_ cells, and orchestrate repression of Th17 differentiation. Previously it was reported that STAT5 regulate T_FH_ cells by binding to the *BCL6* gene promoter and potentially blocking the BCL6 transcription factor (57). BLIMP-1 and BCL6 are mutually antagonistic, and together involved in a regulatory axis that determines differentiation to T_FH_ cells or non-T_FH_ effector CD4 T cells (57, 58). It is well established that during B cell differentiation, the pre-germinal center compartment diminishes, leading to the emergence of three distinct functional subsets: BCL6^hi^ germinal center B cells, BCL6^low^ memory B cells, and BCL6^null^ antibody-secreting cells, which includes proliferating plasma blasts and non-proliferating short-lived plasma cells (59). Genetical ablation of BLIMP-1 on B cells prevented their differentiation into both short-lived and long-lived plasma cells, causing significantly reduced antibody titters (60). We found that B cells in IL-15 stimulated co-cultures exposed to ASC had decreased BCL6 expression with unaffected BLIMP-1 expression, thereby related with a more differentiated B cell phenotype such as plasma cells, memory B cells and MZ B cells. We have previously shown that helminth/TB co-infected individuals, from the same cohort studied herein, have increased levels of total and Mtb-specific IgM despite also having high levels of IgG (25). This correlates with the present finding of IgM^high^ unswitched B cells (the definition of MZ B cells) that increased specifically in helminth-co-infected TB patients.

Our T-B cell co-culture system was intentionally designed as a reductionist model to isolate lymphocyte-intrinsic effects of ASC exposure and T-B interactions, independent of myeloid-derived signals. Although dendritic cells and macrophages are major sensors of helminth antigens *in vivo*, B cells are also professional antigen-presenting cells and express pattern-recognition receptors such as TLR7 and TLR9, enabling them to directly respond to helminth-derived components. Thus, ASC-mediated modulation of B cells and subsequent effects on T cells are biologically plausible in this system. We acknowledge, however, that the absence of innate APCs limits full representation of *in vivo* helminth sensing.

Our findings demonstrate that B cells inhibit T cell proliferation, with helminth antigens exerting species-specific effects on T cell activation. Notably, ASC helminth antigen exposure induced an increase in soluble IL-15Rα and expression of IL-15Rα and IL-15Rβ on T cells, although exhibiting a decreased STAT5 phosphorylation upon rhIL-15 stimulation. There was also a lack of STAT1 (Th1) activation and suppression of STAT3 (Th17) activation. This suggests a novel helminth species-specific modulation of the IL-15 signaling pathway, with long-term effects also on T cell subset differentiation. Future research should aim to elucidate the precise cellular source of cleaved IL-15Rα and further how this can be exploited therapeutically for modulating immune responses in inflammatory diseases like tuberculosis. This dampening in the IL-15/STAT conduit of T cell activation, adds yet another component to, the already complex, helminth-induced regulatory network. It is plausible that this regulation of T cell activation is influenced by B cells. Our clinical data showed an expansion of MZ B cells specifically in helminth-single infected and helminth-co-infected TB patients. The transcriptional profile observed *in vitro* could align with the phenotype of MZ B cells, which are typically BCL6^low^, suggesting a biologically relevant link between helminth antigen exposure and B cell differentiation.

## Data Availability

The original contributions presented in the study are included in the article/**Supplementary Material**. Further inquiries can be directed to the corresponding author.

## Supplementary material

Supplementary Figure 1 Frequency of CD3^+^CD4^+^ T cells were not different among clinical groups presented in **Figure 1**. Frequency of CD3^+^CD4^+^ T cells of total CD3^+^ T cells, from Treg staining in **Figure 1B**, are shown. Scatter plot overlaid on bar graph showing individual data points and bars representing mean ± SEM. Using one-way ANOVA with Dunnett’s multiple comparison test, showed no significant difference.

Supplementary Figure 2 No change in IL-15 receptor expression on CD19+ B cells with the helminth antigen exposure during T and B cell co-cultures. Expression of IL-15Rα (CD215), IL-2/15Rβ (CD122), and IL-2/15Rγ (CD132), on B cells measured four days after co-culture with activated T cells in presence/absence of helminth antigen exposure. Data expressed as mean ± SEM, n=6.

Supplementary Figure 3 STRING-based protein–protein interaction network reveals IL-15 receptor signaling connectivity among immune-regulatory proteins. The network was constructed using the STRING (Search Tool for the Retrieval of Interacting Genes/Proteins) online platform to visualize predicted and known protein–protein interactions among differentially expressed proteins following exposure to *Ascaris lumbricoides* antigen IL-15Rα, TNFB, IL-10Rα, CCL19, IL-2/15Rβ, IL-24, and CXCL11. Nodes represent individual proteins, with node size proportional to the degree of connectivity within the network. Edges denote functional associations supported by experimental data, curated databases, co-expression analyses, and text mining. Edge color reflects the type of evidence contributing to each interaction. This network highlights potential functional relationships among immune-regulatory proteins that are differentially expressed in response to helminth antigen exposure.

Supplementary Figure 4 High Th1 and absence of Th2 surface expression on T cells both before and after rhIL-15 stimulation irrespective of ASC exposure. Flow cytometry was used to determine surface expression of CD183 (Th1), and CD294 (Th2) in rested co-cultures (day 6) (A-B) and 72 hours rhIL-15 stimulated cocultures (C-F).

Supplementary Figure 5 Long-term effect of rhIL-15 stimulation on pSTAT1, pSTAT3, and pSTAT6 in T cells in response to rhIL-15. Phosphorylation of STAT1/3/6 in Th1 polarized T cells by intracellular staining was evaluated by flow cytometry 48 hours after rhIL-15 stimulation of T and B co-culture (A), and after 72 hours (B). Data expressed as mean ± SEM with *, p<0.05, n=6, using one-way ANOVA with Holmsidak comparison test.

Supplementary Figure 6 Prolonged effects of rhIL-15 on STAT1, STAT3, STAT5, and STAT6 phosphorylation in T cells exposed to helminth antigens. Phosphorylation of STAT1, STAT3, STAT5, and STAT6 in CD4^+^ (A), and CD8^+^ (B) T cells was assessed by intracellular staining and flow cytometry, 72 hours after rhIL-15 stimulation of T cell alone. Data are expressed as mean ± SEM, with p < 0.05, n = 4 donors, using one-way repeated measures ANOVA with Dunnett’s multiple comparisons test comparing each antigen condition to T cells alone without rhIL-15 stimulation.
