# Supplementary figures and images for: Ascaris lumbricoides antigen exposure modulates T cell activation via regulation of IL-15Rα expression, STAT5 phosphorylation, and promotes differentiation of BCL6low B cells
Source: Front Immunol. 2026 Feb 23;17:1766483. doi: 10.3389/fimmu.2026.1766483 (PMC12967978; doi:10.3389/fimmu.2026.1766483)

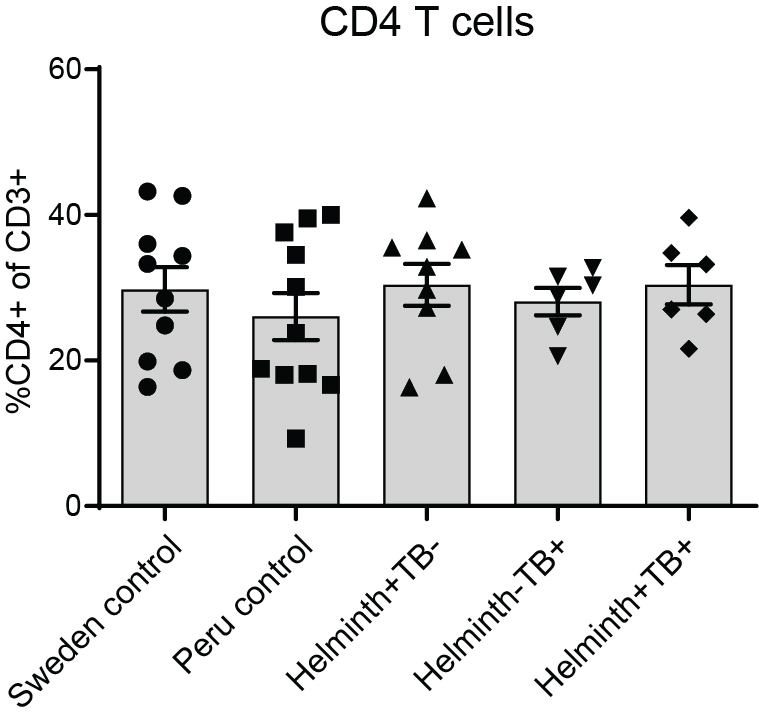

Supplement: Supplementary Figure 1 — Frequency of CD3+CD4+ T cells were not different among clinical groups presented in Figure 1. Frequency of CD3+CD4+ T cells of total CD3+ T cells, from Treg staining in Figure 1B, are shown. Scatter plot overlaid on bar graph showing individual data points and bars representing mean ± SEM. Using one-way ANOVA with Dunnett’s multiple comparison test, showed no significant difference. [file Image1.jpeg]

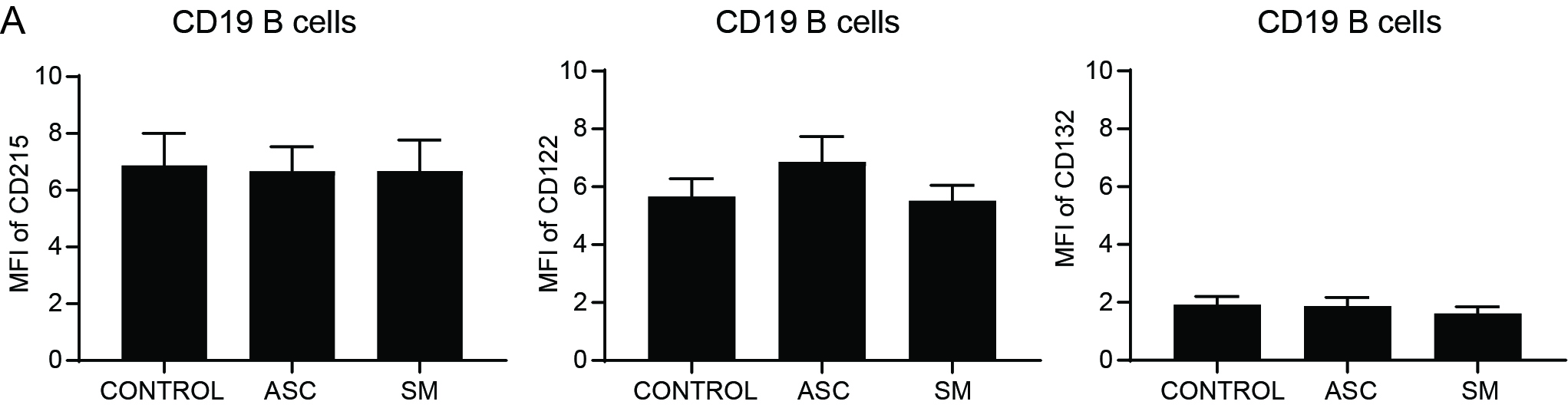

Supplement: Supplementary Figure 2 — No change in IL-15 receptor expression on CD19+ B cells with the helminth antigen exposure during T and B cell co-cultures. Expression of IL-15Rα (CD215), IL-2/15Rβ (CD122), and IL-2/15Rγ (CD132), on B cells measured four days after co-culture with activated T cells in presence/absence of helminth antigen exposure. Data expressed as mean ± SEM, n=6. [file Image2.jpg]

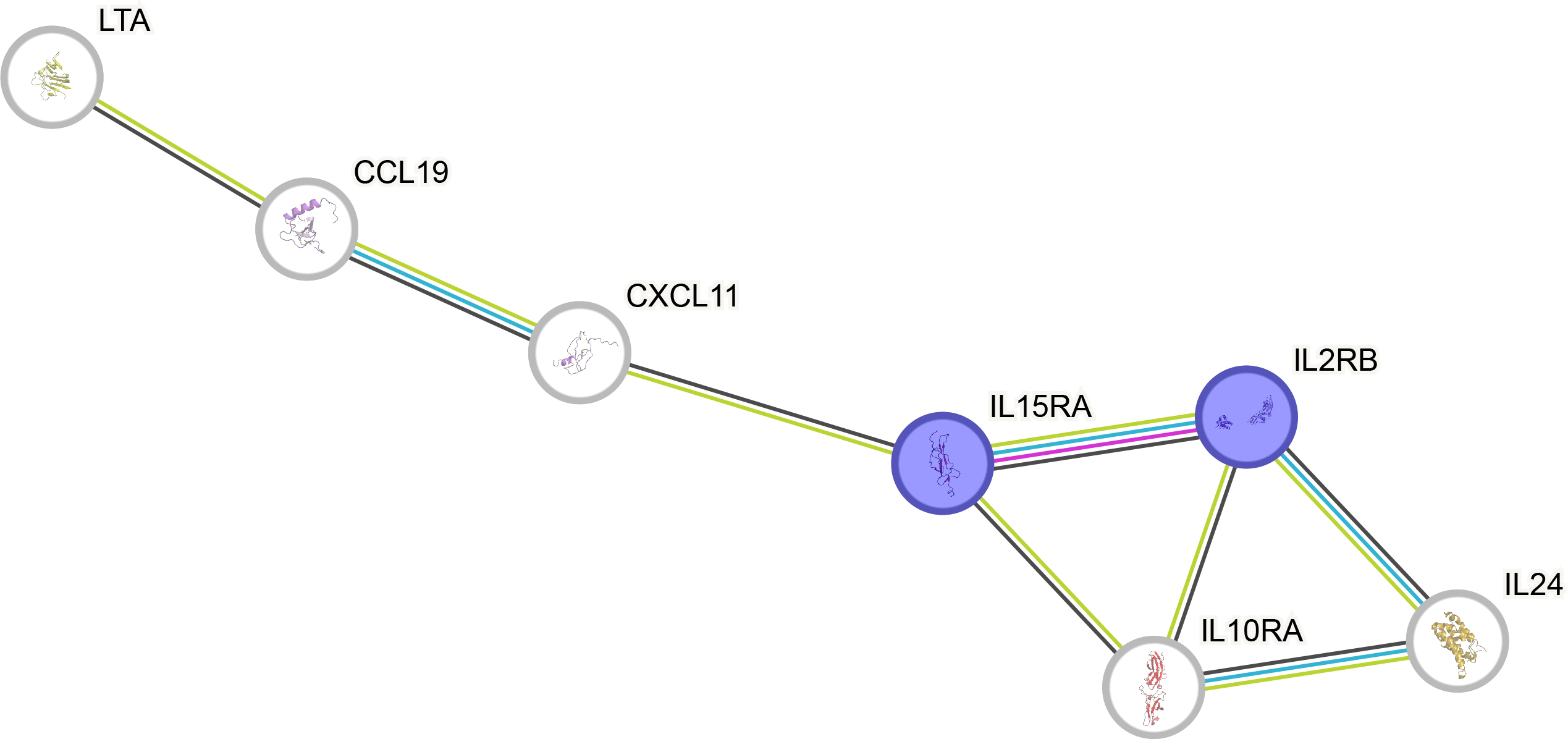

Supplement: Supplementary Figure 3 — STRING-based protein–protein interaction network reveals IL-15 receptor signaling connectivity among immune-regulatory proteins. The network was constructed using the STRING (Search Tool for the Retrieval of Interacting Genes/Proteins) online platform to visualize predicted and known protein–protein interactions among differentially expressed proteins following exposure to Ascaris lumbricoides antigen IL-15Rα, TNFB, IL-10Rα, CCL19, IL-2/15Rβ, IL-24, and CXCL11. Nodes represent individual proteins, with node size proportional to the degree of connectivity within the network. Edges denote functional associations supported by experimental data, curated databases, co-expression analyses, and text mining. Edge color reflects the type of evidence contributing to each interaction. This network highlights potential functional relationships among immune-regulatory proteins that are differentially expressed in response to helminth antigen exposure. [file Image3.jpg]

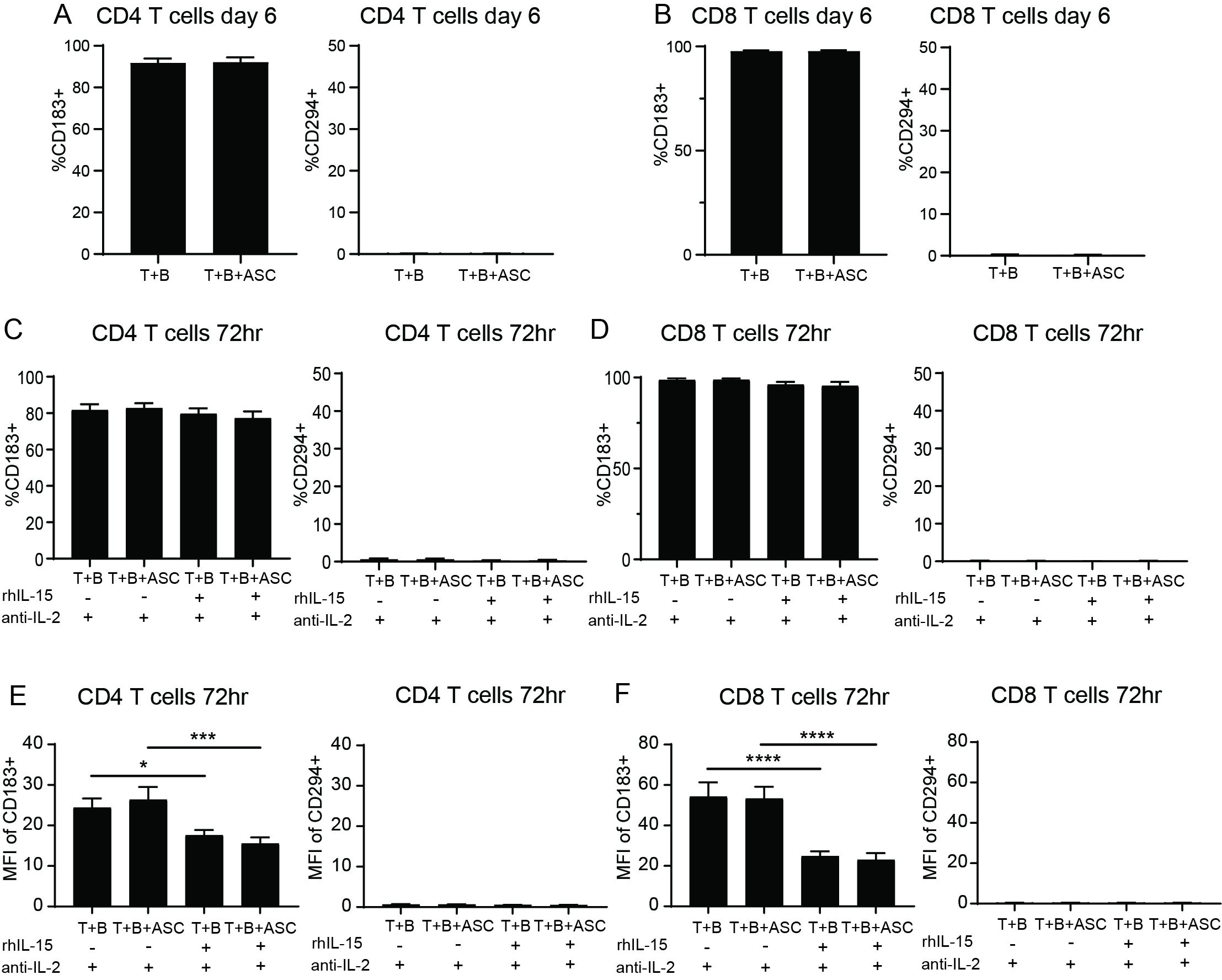

Supplement: Supplementary Figure 4 — High Th1 and absence of Th2 surface expression on T cells both before and after rhIL-15 stimulation irrespective of ASC exposure. Flow cytometry was used to determine surface expression of CD183 (Th1), and CD294 (Th2) in rested co-cultures (day 6) (A-B) and 72 hours rhIL-15 stimulated cocultures (C-F). [file Image4.jpeg]

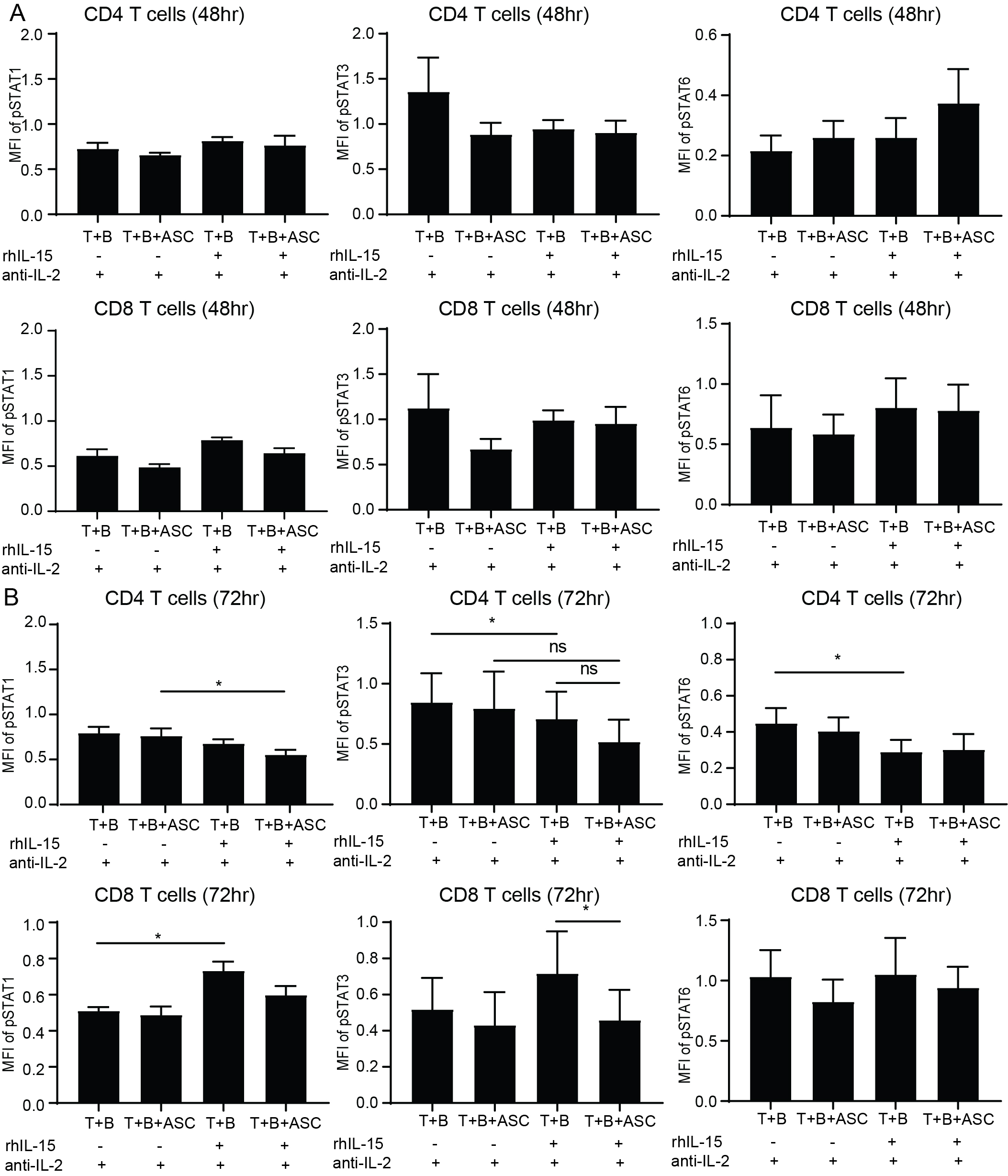

Supplement: Supplementary Figure 5 — Long-term effect of rhIL-15 stimulation on pSTAT1, pSTAT3, and pSTAT6 in T cells in response to rhIL-15. Phosphorylation of STAT1/3/6 in Th1 polarized T cells by intracellular staining was evaluated by flow cytometry 48 hours after rhIL-15 stimulation of T and B co-culture (A), and after 72 hours (B). Data expressed as mean ± SEM with *, p<0.05, n=6, using one-way ANOVA with Holmsidak comparison test. [file Image5.jpg]

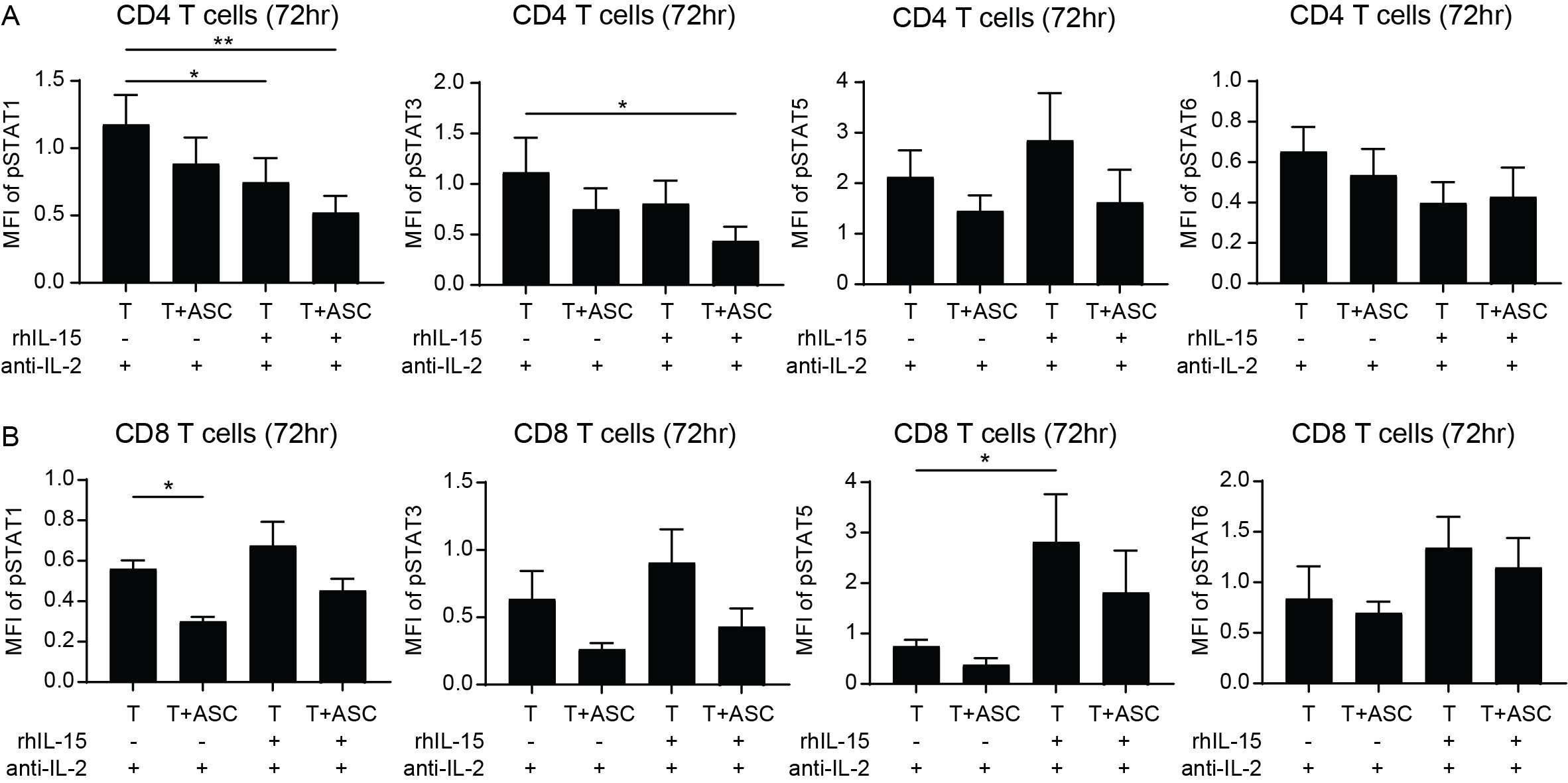

Supplement: Supplementary Figure 6 — Prolonged effects of rhIL-15 on STAT1, STAT3, STAT5, and STAT6 phosphorylation in T cells exposed to helminth antigens. Phosphorylation of STAT1, STAT3, STAT5, and STAT6 in CD4+ (A), and CD8+ (B) T cells was assessed by intracellular staining and flow cytometry, 72 hours after rhIL-15 stimulation of T cell alone. Data are expressed as mean ± SEM, with p < 0.05, n = 4 donors, using one-way repeated measures ANOVA with Dunnett’s multiple comparisons test comparing each antigen condition to T cells alone without rhIL-15 stimulation. [file Image6.jpeg]
